# A voltammetric method coupled with chemometrics for determination of a ternary antiparkinson mixture in its dosage form: greenness assessment

**DOI:** 10.1186/s13065-024-01189-0

**Published:** 2024-05-09

**Authors:** Finan T. Hindam, Basma M. Eltanany, Amal M. Abou Al Alamein, Rasha M. El Nashar, Reham M. Arafa

**Affiliations:** 1 Egyptian Drug Authority, P.O. Box 29, Giza, Egypt; 2 Pharmaceutical Analytical Chemistry Department, Faculty of Pharmacy, Cairo University, P.O. Box 11562, Cairo, Egypt; 3 Chemistry Department, Faculty of Science, Cairo University, P.O. Box 12613, Giza, Egypt

**Keywords:** Antiparkinson drugs, Carbidopa, Entacapone, Differential Pulse Voltammetry, Levodopa, PLS

## Abstract

An electroanalytical methodology was developed by direct differential pulse voltammetric (DPV) measurement of Levodopa (LD), Carbidopa (CD) and Entacapone (ENT) mixture using bare glassy carbon electrode (GCE) in Britton Robinson (BR) buffer (pH = 2.0). A multivariate calibration model was then applied to the exported preprocessed voltammetric data using partial least square (PLS) as a chemometric tool. Additionally, the model was cross-validated and the number of latent variables (LVs) were determined to produce a reliable model for simultaneous quantitation of the three drugs either in their synthetic mixtures or in their marketed pharmaceutical formulation with high accuracy and precision. Data preprocessing was used to tackle the problem of lacking bi-linearity which is commonly found in electrochemical data. The proposed chemometric model was able to provide fast and reliable technique for quantitative determination of antiparkinson drugs in their dosage forms. This was successfully achieved by utilizing sixteen mixtures as calibration set and nine mixtures as validation set. The percent recoveries for LD, CD and ENT were found to be 100.05% ± 1.28%, 100.04% ± 0.53% and 99.99% ± 1.25%, respectively. The obtained results of the proposed method were statistically compared to those of a previously reported High Performance Liquid Chromatography (HPLC) methodology. Finally, the presented analytical method strongly supports green analytical chemistry regarding the minimization of potentially dangerous chemicals and solvents, as well as reducing energy utilization and waste generation.

**Supplementary Information:**

The online version contains supplementary material available at 10.1186/s13065-024-01189-0.

## Introduction

**Fig. 1:**
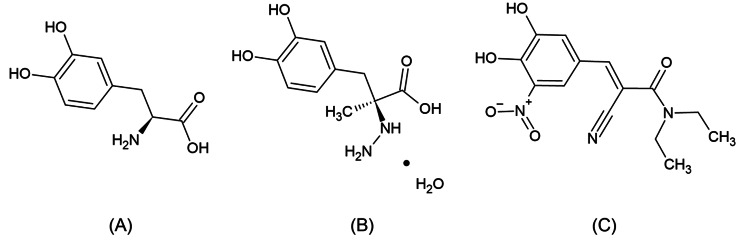
Chemical structures of the three drugs coadminstered for treatment of Parkinson’s disease; LD (**A**), CD monohydrate (**B**) and ENT (**C**)

Parkinson’s disease (PD) is a chronic neurodegenerative disorder characterized by a significant depletion of the dopamine neurotransmitter [1, 2]. Levodopa (LD) is the most successful treatment option for PD symptoms and is commonly regarded as the “gold standard” by which other treatments are evaluated [3]. A major drawback of LD is its liability for metabolism by dopa decarboxylase and catechol-O-methyltransferase enzymes. These enzymes decrease the peripheral LD half-life and its central nervous system (CNS) bioavailability. Accordingly, it is administered in pharmaceutical formulations with enzymatic inhibitory drugs such as Carbidopa (CD) and Entacapone (ENT). LD chemically known as (−)-3-(3,4-Dihydroxyphenyl)-L-alanine [4], is considered as dopamine precursor. CD also known chemically as (−)-L-α-Hydrazino-3,4-dihydroxy-α-methyl hydro cinnamic acid monohydrate [5], is regarded as dopa decarboxylase enzyme inhibitor. Whereas ENT, referred to chemically as (E)-α-Cyano-N, N-diethyl-3,4-dihydroxy-5-nitrocinnamamide [6], is a catechol-O-methyltransferase inhibitor. Demonstration of the chemical structures of the three selected drugs is represented in Fig. 1. Various analytical methods have been developed for the quantitative determination of LD, CD, and ENT simultaneously, separately, or in combination with other medications. These techniques include Liquid Chromatography with tandem mass spectrometry (LC-MS-MS) [7–9], High Performance Liquid Chromatography (HPLC) [10–14], Thin Layer Chromatography [15, 16], spectrophotometry [17–23], capillary electrophoresis [24, 25], and electrochemical methods [26–31]. The majority of chromatographic techniques need high purity solvents including organic solvents, high-cost instrument operation, experienced personnel, long analysis time and sample pretreatment steps [32]. Moreover, spectrophotometric methods possess low sensitivity and selectivity compared to other techniques. On the other hand, electrochemical methods offer numerous advantages compared to the previously mentioned techniques. These advantages include less sample and solvent consumption, affordability, user-friendliness, the ability for electrochemical detection in analytical microsystems or portable devices and short analysis time.

In electrochemistry, two solutions have been presented to resolve the issue of significant overlapping of electrochemical signals obtained from several analytes in a matrix. The first involves the use of modified working electrodes to offer the best possible separation of oxidation peaks [26, 33–35]. However, this modification requires numerous laborious processes that are expensive. The second alternative strategy is to perform multivariate chemometric calibration, either with or without amending the working electrode [36–38].

Recently, chemometric methods have gained a great interest in many analytical techniques including chromatography [39, 40], spectroscopy [41, 42] and electrochemistry [43, 44]. This approach fulfills the requirements of investigating a significant number of mixture components and enables to simultaneously evaluate a number of other variables. The ability to process large amount of data in the form of matrices made the process of data analysis and prediction faster and easier than conservative univariate approaches [45–47, 48].

Considered one of the most environmentally friendly methods of analysis [49], electrochemical detection is accounted for as being simple, rapid, less energy consuming, and not requiring any sample pretreatment [50–52]. Sustainable development, environmental impact and waste minimization are important aspects in green analytical chemistry [53]. The mini potentiostat used in this electrochemical methodology offers a green alternative that reduces danger to users and environment compared to macroscopic systems [54]. Accordingly, this provides endless advantages for consuming less solvents, energy, time and also less waste generation.

The aim of this work is to resolve the ternary mixture of the studied drugs in such a way to obtain a rapid, low cost, accurate and green electroanalytical method based on direct differential pulse voltammetry (DPV) coupled with chemometrics for quantitative determination of LD, CD and ENT in their pure forms, synthetic mixtures and marketed formulations.

Sequential steps were done to achieve the presented regression model. Data preprocessing for peak alignment, baseline correction and smoothing was a crucial step to overcome noise during electrochemical measurements which is common and stated in literature [43, 55]. Model construction and validation were implemented to ensure the validity of the model and its ability of being applied in routine testing.

## Experimental

### Apparatus and software

All voltammetric measurements were achieved using DY 2113 mini potentiostat (Digi-Ivy, Inc., USA) connected to a three-electrode cell, containing a 3-mm CHI-104 GCE (CH Instruments Inc., USA) acting as a working electrode; a 1-mm platinum wire employed as auxiliary electrode, and Ag/AgCl (3 M KCl) electrode representing the reference electrode. The pH measurements were performed using Jenway 3510 digital pH meter (Jenway Instruments, England). Peak alignment and baseline correction were done using MATLAB (R14), while data filtration and chemometric modelling were carried out using SIMCA 14.1.

### Materials and reagents

LD (purity 98.99% ± 0.26%) and CD (purity 100.80% ± 0.15%) both were supplied from Divis Laboratories, India. ENT (purity 100.09% ± 0.35%) was supplied from Hetero labs, India. Stalevo® tablets (purchased from a local market) having a label claim of 200 mg LD, 50 mg CD (as CD monohydrate) and 200 mg ENT per tablet, manufactured by Orion Corporation, Espoo, Finland. All reagents utilized were of analytical grade and bought from Sigma-Aldrich (Germany). Methanol (99%), acetic acid (99.50%), phosphoric acid (85%), boric acid, potassium dihydrogen phosphate, di-potassium hydrogen phosphate and sodium acetate. Britton Robinson (BR) buffer (0.04 M) was prepared by mixing 0.04 M orthophosphoric acid, 0.04 M acetic acid and 0.04 M boric acid. Ultrapure water purified in purelab UHQ (ELGA, United Kingdom) was used throughout this work.

### Standard solutions preparation

Stock standard solutions were freshly prepared in dark containers by weighing and accurately transferring 24.50 mg of pure LD and 22.60 mg of pure CD separately into 50-mL measuring flasks. The volume was completed with BR buffer pH 2.0 to reach a final concentration of 2.50 × 10^− 3^ M and 1.85 × 10^− 3^ M for LD and CD, respectively. For ENT, 30.70 mg of pure drug was weighed and precisely transferred into a 50-mL measuring flask, then the volume was completed with methanol-BR buffer pH 2.0 (50:50, v/v) to reach final concentration of 2.00 × 10^− 3^ M. Subsequent dilutions were done by taking suitable aliquots of the above-mentioned stock solutions and completing the volume with BR buffer pH 2.0 to reach the desired concentrations.

### Experimental conditions

All DPV measurements were accomplished at ambient temperature by implementing the following instrumental conditions for the three drugs: sensitivity, 1000 µAV^− 1^; potential range, from 0.00 to +1.30 V and scan rate of 5 mVs^− 1^. For electrode activation, the surface of working electrode was polished with 0.30 and 0.05 μm alumina slurries [56], then placed in a 0.04 M BR buffer to apply consecutive cyclic voltammetric (CV) sweeps (*n* ≈ 2) having a scan rate of 50 mVs^− 1^ and a potential range from − 0.50 to +1.70 V.

### Data preprocessing

Using MATLAB, useful tools for baseline correction and potential shift correction were implemented and subsequently, the produced pretreated data was then transferred to SIMCA for filtration and Partial Least Squares (PLS) model establishment.

#### Baseline correction

A rapid and flexible baseline fitting algorithm known as Adaptive Iteratively Reweighted Penalized Least Squares (airPLS) [57], was utilized. Several parameters were optimized for achieving baseline correction including number of iterations (15), lambda (10^5^) and the order of the difference of penalties (2). The resulting matrix was then introduced to the next step of data preprocessing.

#### Potential shift correction

Interval Correlation Optimized Shifting algorithm (Icoshift) facilitated synchronization of large amount of data in a matter of seconds by using a piecewise linear correction function depending on an insertion/deletion (I/D) model [58]. The target (reference) signal was the average voltammogram to which all other voltammograms were aligned. On the other hand, the alignment mode was set to split the data into regular intervals of 800 points wide. The final obtained matrix was exported to SIMCA software.

#### SG approach

Data autoscaling followed by SG derivative filtering were done using SIMCA software. Data filtration could be displayed using a set of filter coefficients spread symmetrically around the target data point.

### PLS model design and implementation

A first-order multivariate calibration model was established using Brereton Design including five equally spaced concentration levels for each of the three analytes [59]. The chosen concentration levels meet the criteria of being within the linearity ranges for each specific analyte; based on its previously performed univariate calibration. The design focuses on spanning the mixture space fairly well; so that every level of the five levels is adequately spanned over five different mixtures resulting in 25 mixtures for the whole design.

Multivariate analysis was executed using SIMCA. Data were pareto scaled [60]. PLS regression was carried out for quantification of the three aforementioned drugs. From the twenty-five mixtures, calibration and validation sets were obtained and employed to perform analysis of variance for cross-validated residuals (CV-ANOVA), Permutation test and tests for detection of outliers.

#### Calibration set

Sixteen samples of synthetic mixtures prepared from pure forms of LD, CD and ENT in variable ratios were measured in the potential range from 0.00 to +1.30 V at 5 mV interval, thus acquiring 261 data points. The produced voltammograms were exported in the form of a digital matrix [16 × 261] and then, together with the concentration matrix, were imported to MATLAB for preprocessing steps using airPLS and icoshift. Subsequently, the aligned data was introduced to SIMCA software for applying SG-filter derivatives, PLS and model validation.

#### Validation set

The assay of validation set was done, where voltammetric data of nine laboratory prepared ternary mixtures were recorded and calculated using the same sequential steps and optimized parameters of PLS calibration set.

### Model validation

After building a quantitative model using PLS regression, the model’s quality was examined using coefficient of determination (R^2^) which correlates between nominal and predicted concentrations, test set validation coefficient (Q^2^), root mean square error of cross-validation (RMSECV) [43, 61]. Additionally, the model was evaluated by displaying normal probability plot and also nominal versus predicted concentrations graphical representation. After verification of normal distribution of residuals, CV-ANOVA was applied to evaluate the validity of the model.

### Analysis of pharmaceutical formulation

Twenty film coated Stalevo® tablets were finely ground and a quantity corresponding to 200 mg LD, 50 mg CD and 200 mg ENT was accurately transferred to 250-mL measuring flask using methanol-BR buffer pH 2.0 (50:50, v/v) as a diluent. Sonication for 20 min then filtration was performed to get a clear solution. To obtain the desired concentration, further dilution was done following the same procedure as previously mentioned under Sect. (2.3. Standard solutions preparation).

## Results and discussion

### Experimental conditions enhancement

Depending on the previously reported methods, electrochemical behavior of the three studied drugs was illustrated in various supporting electrolytes including: phosphate buffer [37, 62, 63], Mcllvaine buffer [64], BR buffer [35], perchloric acid [38], acetate buffer [65], and sulfuric acid solution.

Practically, choosing the supporting electrolyte was accomplished after trying several buffers including: acetate buffer, phosphate buffer and Britton-Robinson buffer at different pH values taking into consideration physicochemical parameters of the studied drugs. The pKa values of the three drugs are: 2.3, 3.59 and 4.5 for LD, CD and ENT, respectively [66, 67]. Better solubility and stability conditions were achieved at low pH values. In neutral and alkaline solutions, the drugs were unstable due to autoxidation [68, 69]. Additionally, acidic conditions are considered optimum for creating both maximal and stable signals [35, 37, 38]. For this reason, BR buffer was used at pH 2.0 in all preparations of stock standard solutions and their subsequent dilutions except for ENT stock standard solution due to its limited solubility in aqueous solutions [70]. To resolve this issue a mixture of BR buffer and methanol (50:50, v/v) was utilized to prepare only the stock standard solution and the subsequent dilutions were proceeded as previously mentioned.

DPV is a valuable chemical analysis technique with practical scientific applications due to its great sensitivity. Pulsed-voltammetric methods are very effective because they record the faradaic current soon after the potential is altered, enabling the background current to become equalized and improving the signal to noise ratio [71]. While Square Wave Voltammetry offers certain advantages over DPV, such as shorter analysis times and slightly better sensitivity, DPV is more suitable for irreversible oxidation or reduction systems owing to its slow kinetics of electron transfer at electrode surface [72]. Both Carbidopa and Entacapone exhibit irreversible oxidation behavior at the surface of GCE, while Levodopa shows a reversible oxidation one [34, 64, 73].

**Fig. 2:**
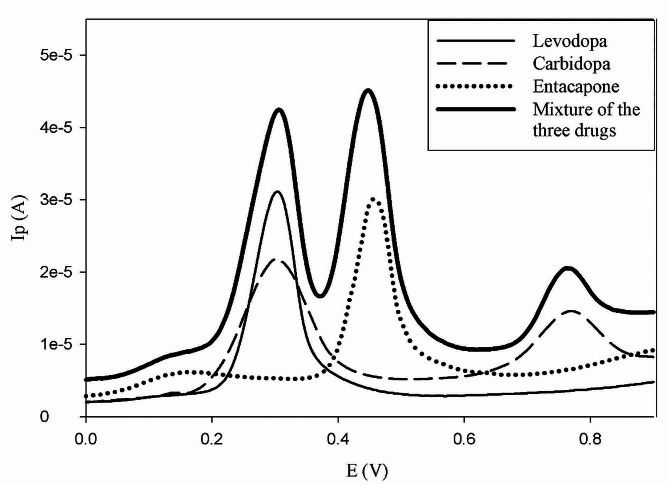
Differential pulse voltammograms of each drug; LD, CD and ENT at concentration levels 4.0 × 10^− 4^ M, 2.78 × 10^− 4^ M and 1.0 × 10^− 4^ M, respectively, and their representative mixture, at the same concentration levels

Electrode activation is a crucial step after each DPV measurement. This is attributed to the strongly observed adsorption of the studied drugs on the GCE surface, similarly happens to other drugs belonging to the class of phenolic, catecholics and gallate derivatives [38]. Figure 2. represents the overlapping signals of the three drugs when measured simultaneously by DPV using the aforementioned supporting electrolyte.

### Data preprocessing

After DPV measurements of 25 samples, the voltammograms revealed two additional complications besides the presence of severe overlapping of analytical profiles; which were (1) baseline drift and (2) potential shifts in oxidation peaks. Both problems are considered sources of data bi-linearity and data preprocessing was a critical step before applying the PLS model in order to achieve more accurate and precise results.

#### Baseline correction

**Fig. 3:**
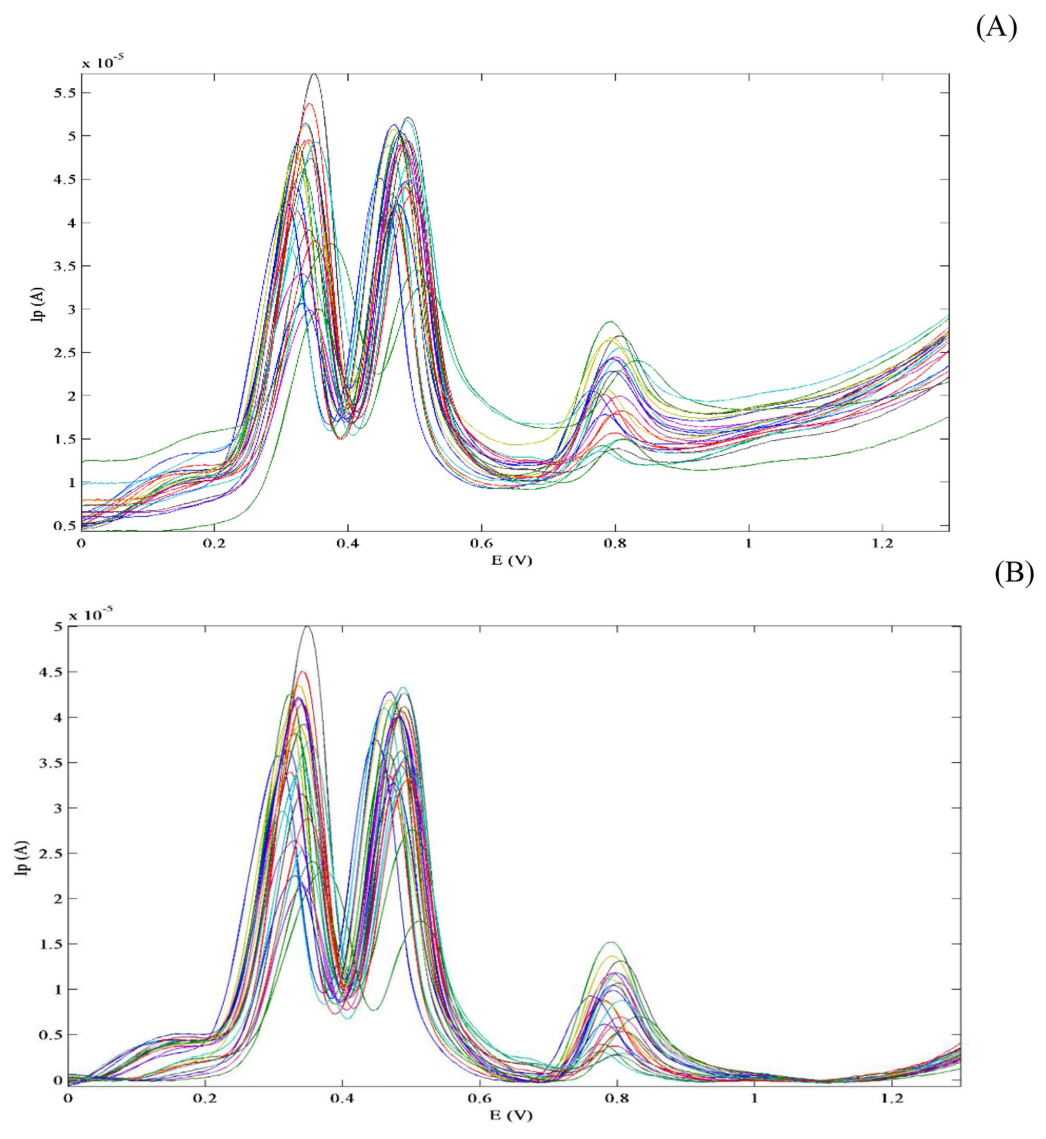
Representative voltammograms after baseline correction (**A**) Raw data (**B**) After applying airPLS function in Matlab

Baseline drift, background fluctuations and noise could have a negative impact on qualitative or quantitative analytical outputs. For this reason, baseline was fitted and corrected using airPLS. As the algorithm runs, the Sum Square Errors (SSE) between the adjusted baseline and authentic signals are iteratively changed, and the weights of the SSE are adaptively gained using the subtraction between the formerly adjusted baseline and the authentic signals [57]. The net result was gradual approximation of a complex baseline as demonstrated in Fig. 3.

#### Potential shift correction

**Fig. 4:**
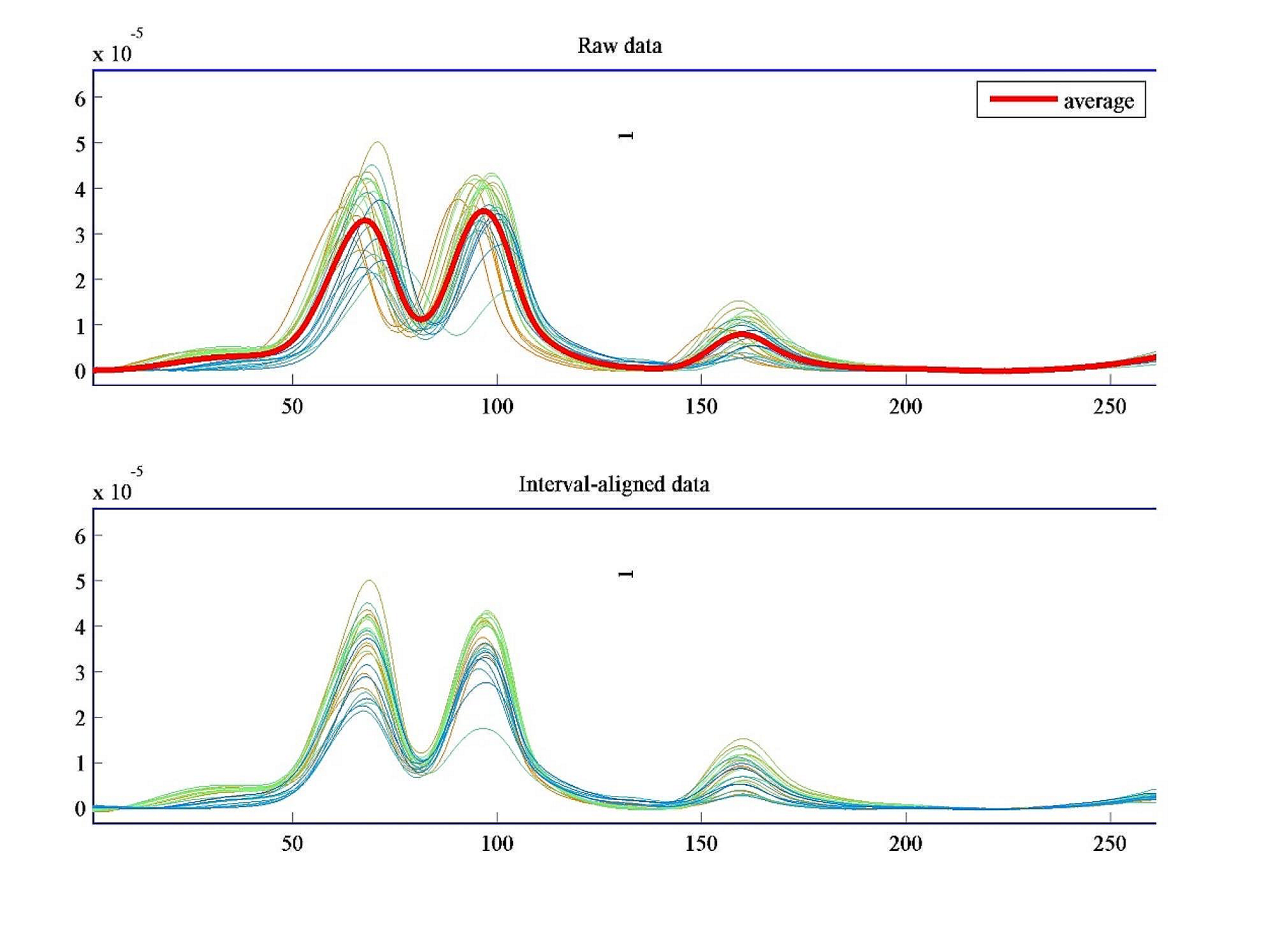
Alignment of voltammograms towards a target signal (average) using Icoshift function in Matlab

The presence of peak-to-peak shift has been recognized during voltammetric measurements from run to run. In literature, several factors were mentioned to contribute to the occurrence of this phenomenon. These include: adsorption of analytes on electrode surface, pH variations or composition of sample solution being fluctuated [74]. As a general rule, non-linear signals typically result in shifting, broadening or increasing in signal amplitude; any of these obstacles renders it difficult to use multilinear data processing algorithms properly. To overcome this drawback, several trials were done to reach the optimum alignment of peaks by choosing the target signal and the number of intervals. As a consequence, the fragments in the sample to be aligned were moved in order to reach their maximum cross-correlation in accordance with the target fragment [58]. Finally, all DPV signals were aligned and shifted towards a target signal which was the average voltammogram as seen in Fig. 4.

#### SG approach

SG derivatives are used mainly to reduce scatter effects of continuous spectra (voltammograms in this case); this is done by fitting a low degree polynomial function to the data in a piecewise manner. Then computing the first and second derivatives from the generated polynomial at points of interest. Removal of an additive baseline (offset) and linear baseline were the main purposes for first and second derivatives respectively [75].

Derivatization may produce very noisy derivative spectra due to signal reduction. SG utilized a combination of smoothing and averaging to reduce the instrument time needed, allowing data convolution to be performed using as suitable computer software. As a result, the noise is reduced by approximately the square root of the number of points used [76].

Generally, the sources of noise impacting electrochemical data are: low-frequency signals, quasi-random noise with high-frequency, spikes possessing short pulses with high amplitude or drift in baseline. SG-filter was used in this case as an algorithm based on smoothing to eliminate the noise and enhance the signals.

### Multivariate data analysis

**Fig. 5:**
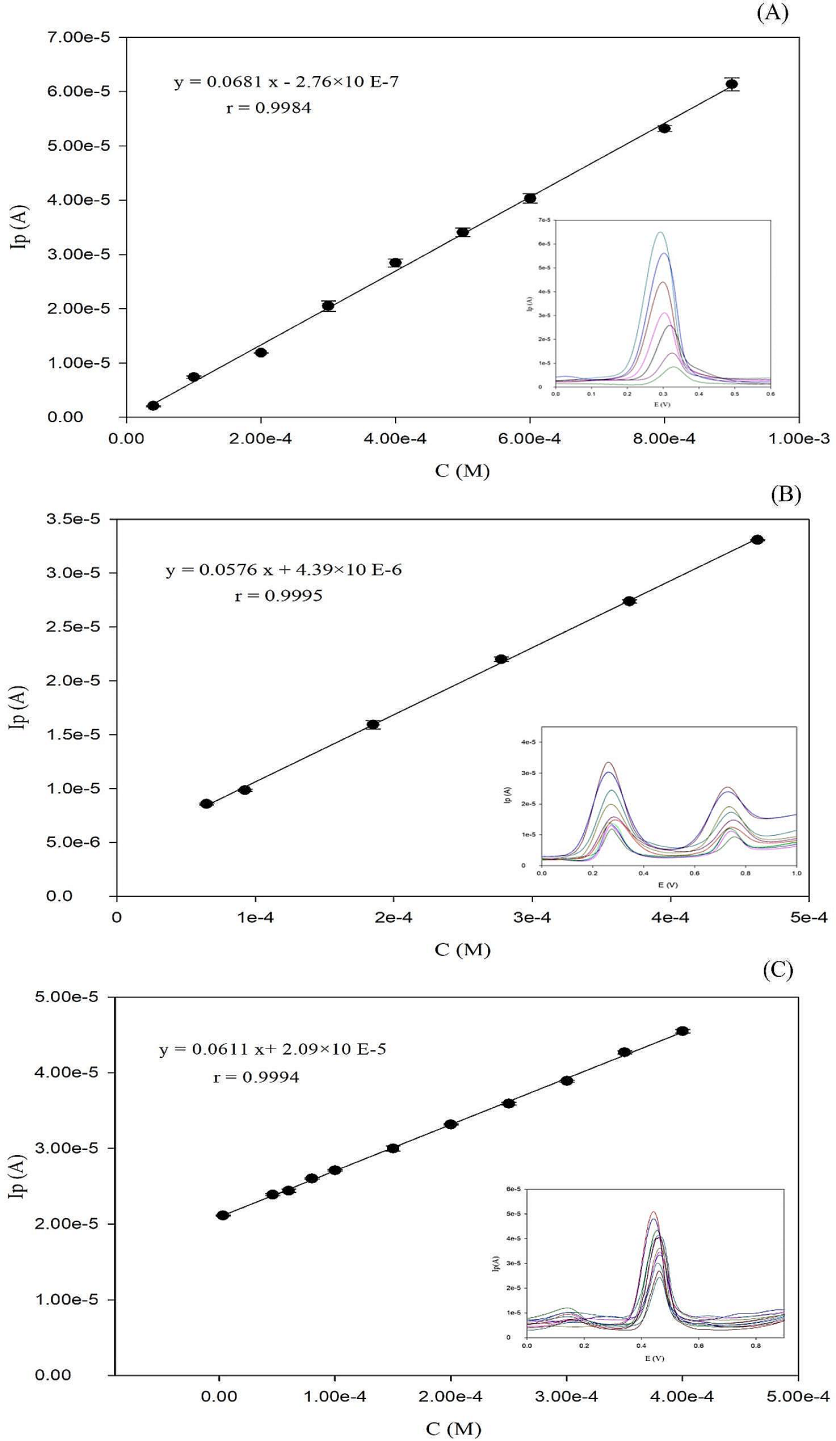
Calibration curves of LD (**A**), CD (**B**) and ENT (**C**) using DPV technique at different concentrations. Inset: Representative differential pulse voltammograms of the three drugs; LD, CD and ENT, respectively

Practically, univariate calibration was performed for each individual drug before application of multivariate analysis, to ensure the linearity of concentration levels introduced into the model. The linearity ranges were: LD (4.0 × 10^− 5^ to 9.0 × 10^− 4^ M), CD (6.48 × 10^− 5^ to 4.63 × 10^− 4^ M) and ENT in the range (3.20 × 10^− 6^ to 4.0 × 10^− 4^ M). The obtained results for each drug are presented in Fig. 5. and Table 1, respectively.

**Table 1:** Regression data of the calibration lines for quantitative determination of each drug of LD, CD and ENT, by DPV.

Parameter	LD	CD	ENT
Anodic peak potential Ep (V)	0.29 ± 0.059	0.28 ± 0.017	0.46 ± 0.011
Linear range (M)	(4.00 × 10^− 5^) - (9.00 × 10^− 4^)	(6.48 × 10^− 5^) - (4.63 × 10^− 4^)	(3.20 × 10^− 6^) - (4.00 × 10^− 4^)
Intercept	− 2.76 × 10^− 7^	4.39 × 10^− 6^	2.09 × 10^− 5^
Slope	0.0681	0.0576	0.0611
Correlation coefficient (r)	0.9984	0.9995	0.9994

One of the most frequently used tools in multivariate calibration methodologies is PLS. It exhibits a variety of applications in instrumental analysis including: chromatographic, spectroscopic and electrochemical techniques [77]. Generally, two steps are needed to construct the multivariate calibration model. The first step is calibration, where a relation is established between the instrumental signal (i.e., voltammograms) and the corresponding component concentrations from a group of standard samples, while the second step is prediction, where the results obtained from calibration step are utilized to estimate concentrations of components in unknown sample mixture. PLS is viewed as a factor-based multivariate calibration methodology. It has the majority of the benefits of the classical least squares (CLS) approach. Furthermore, it retains the inverse least squares (ILS) advantage of performing the analysis of a single chemical component at a time while avoiding the ILS limited selection problems [77]. In PLS, both response matrix and concentration matrix are used in modeling. Additionally, PLS maximizes the covariance between the two matrices by determining a set of regression coefficients that correlate between both dependent and independent sets.

#### Choice of ideal number of latent variables

**Fig. 6:**
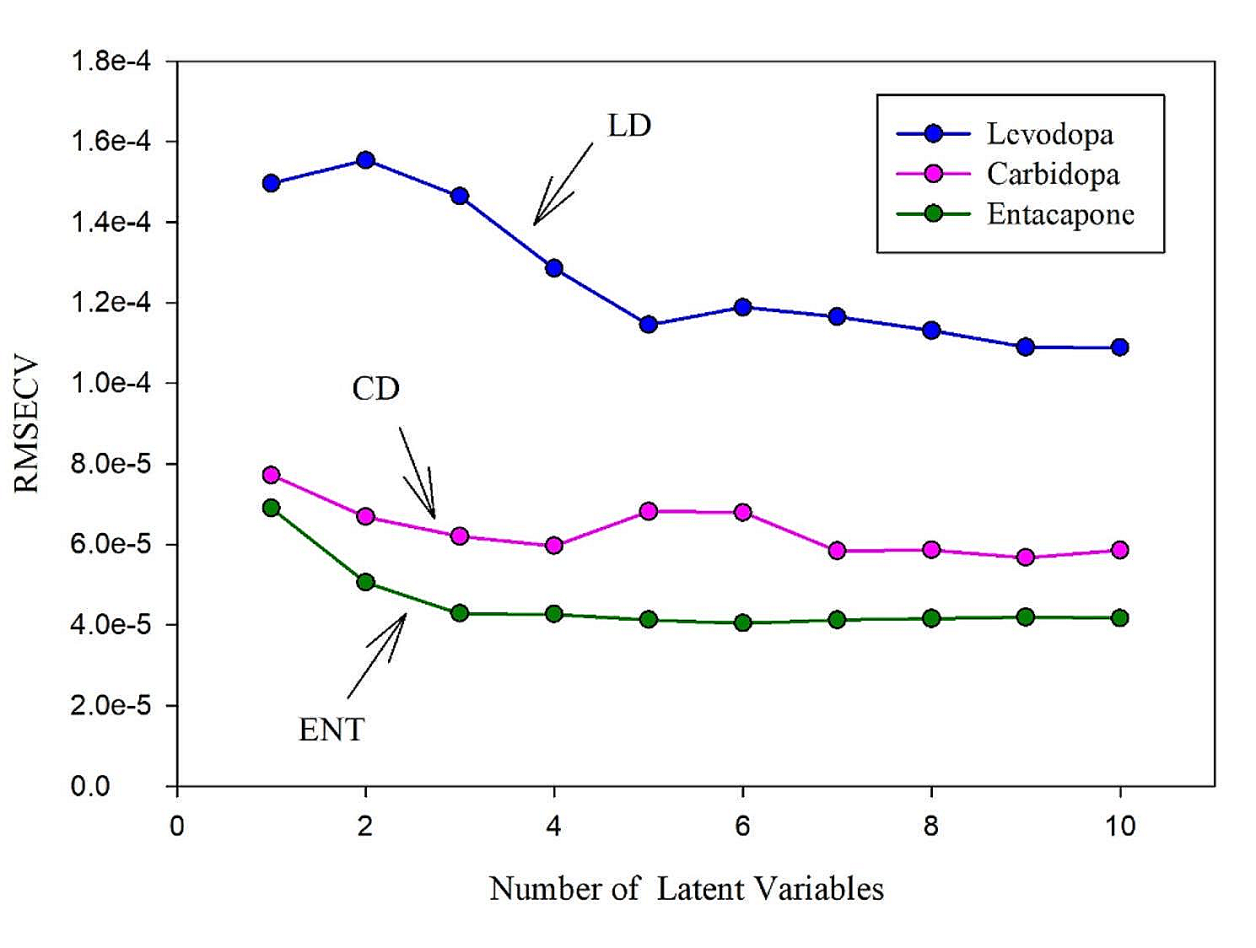
RMSECV plot of the cross-validation results for the calibration set as a function of the number of latent variables used to construct PLS calibration model for LD, CD and ENT.

The ideal number of latent variables (LVs) selected is an influential step for achieving correct quantitation during PLS calibration. In case of selecting a number of LVs more than the demanded number, higher noise would be computed into the constructed calibration model. Furthermore, selecting a number that was too small, would result in omitting significant data which could be essential for the calibration. One way to optimize the number selected, such that the RMSECV of the least number of factors for that model wasn’t significantly greater than the RMSECV of the model with an additional factor [78]. This way reduces the potential of overfitting the calibration data. Consequently, the ideal number of LVs chosen was found to be seven factors as demonstrated in Fig. 6.

#### Model validation

Validation of the previously developed model was a key step in demonstrating that the model’s predictions were accurate and its performance is adequate. The chemometric strategies that are used to evaluate a predictive model’s quality are indicated as validation [79]. One way to perform this is defining a diagnostic metric; it could be based on either the calculation of residuals or a model parameter [43].

#### Cross-validation ANOVA

CV-ANOVA, one of the most essential tools to assess the robustness of PLS models, is based on predictive residuals that are cross-validated [80]. CV-ANOVA tests for significance of equal cross-validated predictive residuals (Q^2^Y_CV_) of two models being compared using the F-distribution. Normal distribution of residuals of the two compared models is the main assumption of F-test. CV-ANOVA results are illustrated in Table 2., where the mean squares (MS), or variances resulted by dividing each quantity of Y of the training set (SS) by the respective degrees of freedom (DF). The F-test depends on the ratio of MS regression to MS residual and the p-value denotes the probability level for a model with a specified F-value being an accidental outcome.

**Table 2:** Results for cross validation-ANOVA

Parameter	SS	DF	MS	F	*p*	SD
**LD**						
Total corrected ^a^	24	24	1			1
Regression ^b^	10.54	20	0.527	0.157	0.998	0.726
Residual ^c^	13.46	4	3.37			1.83
**CD**						
Total corrected	24	24	1			1
Regression	19.81	20	0.991	0.947	0.596	0.995
Residual	4.19	4	1.05			1.02
**ENT**						
Total corrected	24	24	1			1
Regression	16.29	20	0.815	0.423	0.912	0.903
Residual	7.71	4	1.93			1.39

^a^ Total corrected: the quantities (SS) of the Y of the training set corrected for the mean

^b^ Regression: fraction of total corrected SS estimated by Cross-validation and accounted for by the PLS model

^c^ Residual: difference between total corrected and regression SS; it is the fraction of total corrected unaccounted for by the PLS model

#### Premutation test

This tool also enables estimation of PLS model significance. This was done primarily by estimation of the model and its R^2^Y and Q^2^Y, then X-variables are fixed and the elements in the Y-vector are interchanged randomly a certain number of times [80]; in this work around 40 permutations were done. For each permutation executed a new PLS model was formed as a result of fitting X and permuted Y leading to a reference distribution of R^2^Y and Q^2^Y. The outcome of all new models is then employed to prove the statistical significance in accordance with R^2^Y and Q^2^Y parameters of the original PLS model (with non-permuted Y) as illustrated in Additional file 1: Fig. S1. Taking into consideration that the original model’s R^2^Y and Q^2^Y are consistently higher than the relevant values of the models fitted to the permuted responses. The intercept value approaching zero (R^2^ = 0.35) and the negatively signed intercept (Q^2^ = -0.93) supports the validity of the PLS model.

#### Model efficiency and predictability

Assessment of the efficiency of the developed model strongly depends on calculating RMSECV using the following illustrated Eqs. (1) and (2), and presented in Table 3. Strong evidence for enhancing model predictability was also proved by calculating root mean square error of prediction (RMSEP) and its relevant percentage at each step of data preprocessing as provided in Table 4.

**Table 3:** Statistical results of the PLS model for the determination of LD, CD and ENT in laboratory prepared mixtures by DPV

Parameter	PLS		
	LD	CD	ENT
RMSECV ^a^ (M)	1.17 × 10^− 4^	5.83 × 10^− 5^	4.12 × 10^− 5^
R^2 b^	0.9993	0.9984	0.9940

^a^ for the calibration set

^b^ for linear regression between nominal and predicted concentrations in both calibration and validation sets

**Table 4:** Calculated RMSEP and its relevant percentage for the studied drugs at each data preprocessing step

		Raw data	
	LD	CD	ENT
RMSEP	6.02 × 10^− 5^	4.36 × 10^− 5^	2.91 × 10^− 5^
REP%	14.5	12.43	11.89
		After airPLS	
RMSEP	1.04 × 10^− 4^	6.03 × 10^− 5^	4.27 × 10^− 5^
REP%	25.83	18.14	17.85
		After Icoshift and airPLS	
RMSEP	4.27 × 10^− 5^	2.71 × 10^− 5^	1.83 × 10^− 5^
REP%	10.94	7.8	7.57
		After SG	
RMSEP	1.98 × 10^− 6^	4.28 × 10^ − 6^	2.11 × 10^− 6^
REP%	0.48	1.19	0.85

1 $$\varvec{R}\varvec{M}\varvec{S}\varvec{E}=\sqrt{\frac{{\sum }_{\varvec{i}=1}^{\varvec{n}}{\left({\varvec{y}}_{\varvec{p}\varvec{r}\varvec{e}\varvec{d}}-{\varvec{y}}_{\varvec{a}\varvec{c}\varvec{t}}\right)}^{2}}{\varvec{n}}}$$

2 $$\varvec{R}\varvec{E} \left(\varvec{\%}\right)=\frac{100}{{\varvec{y}}_{\varvec{m}\varvec{e}\varvec{a}\varvec{n}}}\varvec{*}\sqrt{\frac{{\sum }_{\varvec{i}=1}^{\varvec{n}}{\left({\varvec{y}}_{\varvec{P}\varvec{r}\varvec{e}\varvec{d}}-{\varvec{y}}_{\varvec{a}\varvec{c}\varvec{t}}\right)}^{2}}{\varvec{n}}}$$

Where the actual (nominal) and predicted concentrations of each drug are denoted by y_act_ and y_pred_, respectively, n stands for the number of samples in the calibration or validation set and y_mean_ is the average of all nominal concentrations in the same set.

#### Nominal versus predicted concentration

**Fig. 7:**
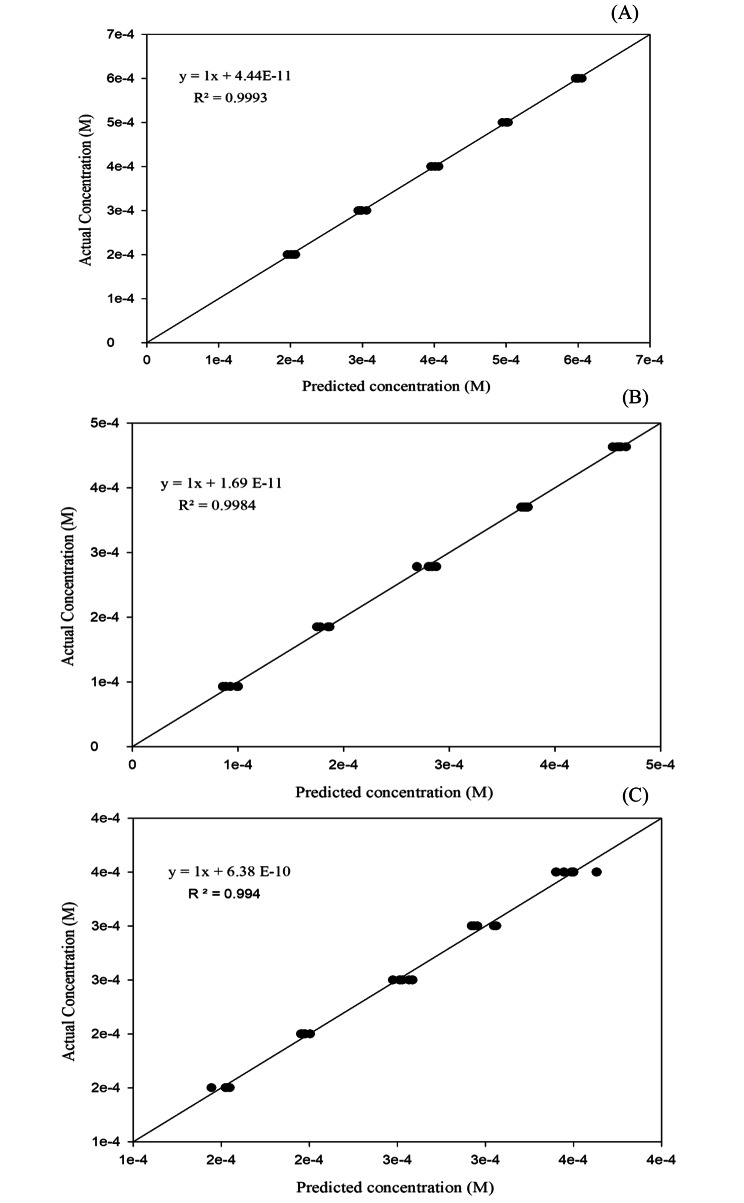
PLS correlation plot of the actual versus predicted concentrations for LD (**A**), CD (**B**) and ENT (**C**) for both calibration and validation sets

Plotting the nominal (true) concentrations of the calibration and validation samples against the predicted concentration values as expressed in Fig. 7. The coefficient of determination (R^2^) was also evaluated to demonstrate the predictive capability of the model as shown in Table 3. The resulting values of R^2^ indicated a strong correlation between the predicted and observed values.

#### Diagnostic tools for detection of outliers

Outliers cause errors or unexpected variability in particular observations; several reasons were reported for this phenomenon including instrumental errors or drift or samples from other populations than expected [75].

#### Leverage

Leverage is a metric for the impact of an observation on PLS model. Being proportional to Hotelling’s T^2^, leverage simply estimates how far a data point is from the model’s center. High leverage observations could have a significant effect on the model; those that fall close to a component axis support the model while others that fall far from the component line cause the model to rotate.

In SIMCA software, the observed leverages in the X and Y spaces are calculated as the diagonal elements of the matrices H_0_ and H_y_, respectively, as illustrated in Eqs. (3) and (4):

3 $${\mathbf{H}}_{0}=\mathbf{T}{\left({\mathbf{T}}^{\mathbf{{\prime }}}\mathbf{T}\right)}^{-1}{\mathbf{T}}^{\mathbf{{\prime }}}$$

4 $${\mathbf{H}}_{\mathbf{y}}=\mathbf{U}{\left({\mathbf{U}}^{\mathbf{{\prime }}}\mathbf{U}\right)}^{-1}{\mathbf{U}}^{\mathbf{{\prime }}}$$

#### Residual normal probability plot

**Fig. 8:**
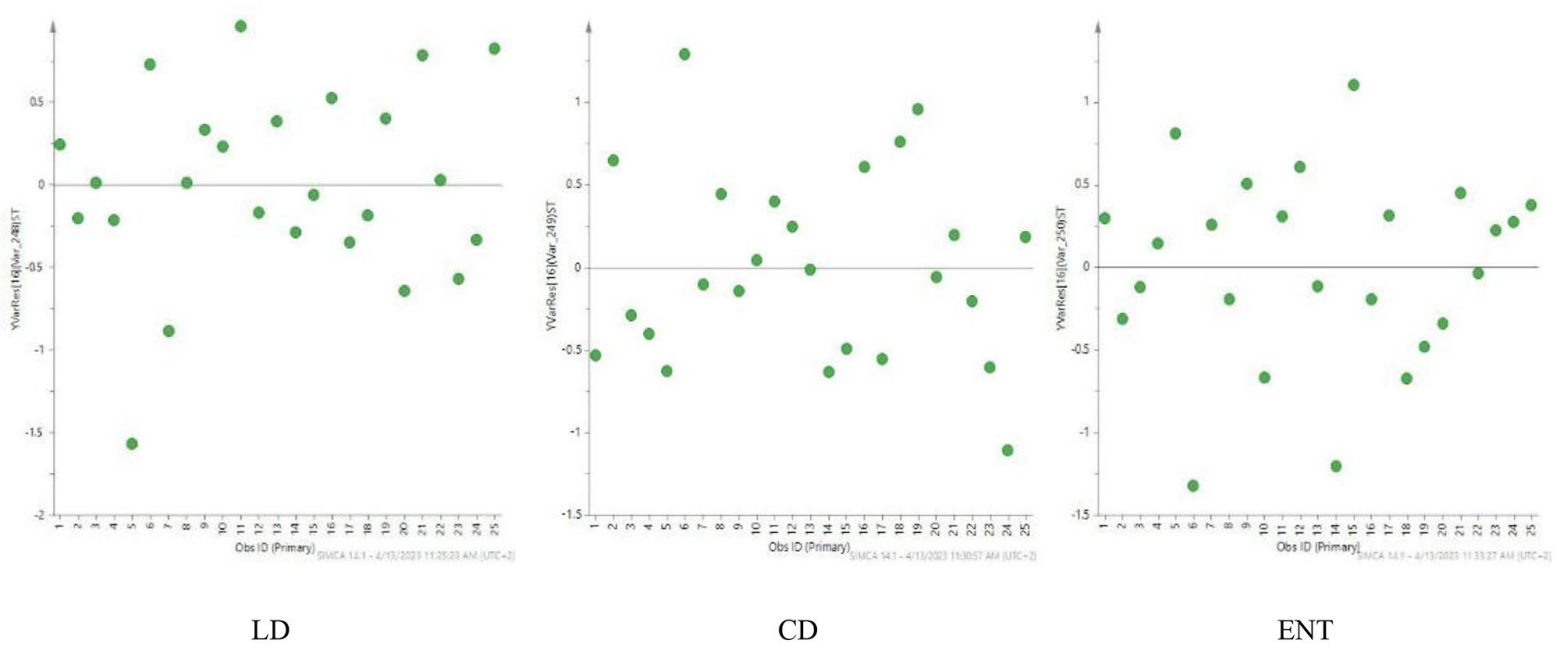
Normal probability plot for the three drugs; LD, CD and ENT. Applying the constructed PLS calibration model

For each drug a normal probability plot of residuals is displayed in Fig. 8. The residuals were normalized on a double Log scale and calculated by dividing the raw residual by the residual standard deviation (RSD). Accordingly, detection of outliers and assessment of residuals’ normality were done using this graphical presentation. As demonstrated, the obtained residuals are normally and randomly distributed since all points are falling between − 2 and + 2 standardized standard deviations. Any point lying outside this specified range is considered an outlier.

#### Hotelling (T^2^)

Hotelling’s T^2^ plot shows the gap between each selected observation and the score space. T^2^ range is determined as the total of the squared scores divided by their squared standard deviations over the given range of components. Additional file 2: Fig. S2. shows T^2^ computed for the range of the chosen components. Knowing that for a specific observation a high T^2^ range value, i.e., a value significantly higher than the critical thresholds, denotes that the observation exists apart from other observations in the chosen range of components in the score space. Therefore, this is considered a potential outlier that, if found in the training set, could negatively impact the model’s performance.

### Analysis of pharmaceutical formulation

The same data preprocessing procedures (previously discussed under Sect. (2.5. Data preprocessing)) were achieved on the resulting voltammograms from tablets measurement and pre-treated data was then introduced to this model with the same previously mentioned parameters. The applied method for pharmaceutical formulation containing the three drugs revealed results of good recovery and precision, Table 5.

**Table 5:** Results for determination of LD, CD and ENT in Stalevo® tablets by the proposed method

Parameter	PLS		
	LD	CD	ENT
R^n^ ± SD	99.58 ± 0.83	101.26 ± 0.55	99.02 ± 0.52

^n^ for three determinations

### Greenness comparison between the suggested voltammetric method and the reported HPLC method

Determining the environmental impact of numerous analytical techniques in relation to their complying to the greenness chemistry theory was essential. This was achieved by evaluating four main criteria: enormous quantities and hazards associated with chemical use, high energy utilization, occupational hazards and waste production [81].

#### Analytical Eco-scale

Eco-scale aspect is an optimum tool to compare and choose the greenest option for analysis [82]. It uses a total of 100 penalty points. For each analytical method, the total penalty points were calculated and subtracted from a base of 100 [81]. The higher the score being accomplished, the eco-friendlier and more cost-effective the analytical method will be [83, 84]. The resulting eco-scale score was found to be 90 and 77 for the suggested voltammetric and reported HPLC methods respectively as demonstrated in Table 6. This manifests that the proposed voltammetric approach is more environmentally friendly than the reported HPLC method.

**Table 6:** Penalty points (PPs) for the proposed and reported methods

Parameters	Penalty points (PPs)	
	Proposed method	Reported method [12]
**Reagents**		
Britton- Robinson buffer	1	-
Methanol	6	-
Water	0	0
Tetrahydrofuran	-	6
Trifluoracetic acid	-	4
Acetonitrile	-	4
**Instrument**		
Energy	0 (≤ 0.1 kWh per sample)	1 (> 0.1 kWh per sample)
Occupational hazard	0	3
Waste	3	5
**Total PPs**	Ʃ 10	Ʃ 23
**Analytical Eco-scale score**	90 Great green analysis	77 Great green analysis

Analytical eco-scale score = 100 (the ideal score for green analytical method)

Analytical eco-scale score > 75 (a great green analysis)

Analytical eco-scale score 50–75 (green analysis is acceptable)

Analytical eco-scale score ˂ 50 (green analysis is inadequate)

### Statistical evaluation for both the suggested method and the reported method

The proposed voltammetric method expressed both accuracy and precision resulting in good recoveries for LD, CD and ENT in their pure forms, which were statistically compared with the reported HPLC technique [12] as illustrated in Table 7. This table reveals that the calculated t-test and F values are less than the tabulated ones. Consequently, the null hypothesis is maintained; as there is no significant difference between the suggested method and the reported one.

**Table 7:** Statistical comparison between the results of the proposed voltammetric method and the reported HPLC method for determination of LD, CD and ENT in their pure forms

Parameter	LD		CD		ENT	
	Proposed voltammetric method	Reported HPLC method [12]	Proposed voltammetric method	Reported HPLC method [12]	Proposed voltammetric method	Reported HPLC method [12]
Mean	99.76	100.13	100.69	100.78	99.50	99.63
SD	0.79	0.65	1.13	0.71	0.52	0.47
Variance	0.62	0.42	1.28	0.51	0.27	0.22
n	5	5	5	5	5	5
t−test^b^	-0.81		-0.15		− 0.41	
F value ^b^	1.48		2.51		1.23	

^a^ Reported HPLC method was performed using C8 (150 × 4.0 mm, 5 μm) column as a stationary phase, isocratic elution using mobile phase containing buffer solution and acetonitrile (75: 45, v/v). The buffer solution contained bi-distilled water, tetrahydrofuran and trifluoracetic acid (970: 30: 0.2, by volume). The flow rate was 1.0 mL/min and the detection was carried out at 282 nm for LD, CD and ENT (12)

^b^ Values of calculated* t*-test and f-value between the proposed method and reported HPLC method where t-tabulated at *p* = 0.05 (2.132) and F-tabulated at *p* = 0.05 (6.39)

## Conclusion

The direct voltammetric measurement with bare GCE in the proposed method proved to be sufficient for obtaining signals that can be easily interpreted by PLS approach, allowing simultaneous determination of LD, CD and ENT in a single measurement without additional steps or time consumption. Furthermore, data preprocessing procedures obviously decreased sources of variations during performing multivariate calibration in comparison with raw data, this enables better predictability of future samples using this ternary calibration model. The results of accuracy and precision obtained when applying the developed method were satisfactory in accordance with the reported HPLC method. On the other hand, the suggested technique would be a safe alternative for routine testing of the pharmaceutical combination under study, especially in laboratories lacking more advanced analytical instruments. Finally, the developed model provides a simple, rapid, affordable, reliable and environmentally friendly method for routine analysis of LD, CD and ENT simultaneously in their pure forms, synthetic mixtures and pharmaceutical dosage forms.

### Electronic supplementary material

Below is the link to the electronic supplementary material.

Supplementary Material 1

Supplementary Material 2

## Data Availability

All data as well as a software application utilized during this study are included in this article and supplementary materials.
