# Supplementary material for: A voltammetric method coupled with chemometrics for determination of a ternary antiparkinson mixture in its dosage form: greenness assessment
Source: BMC Chem. 2024 May 9;18(1):90. doi: 10.1186/s13065-024-01189-0 (PMC11080133; doi:10.1186/s13065-024-01189-0)
Supplement: Supplementary file 1 — Supplementary Material 1 [file 13065_2024_1189_MOESM1_ESM.docx]

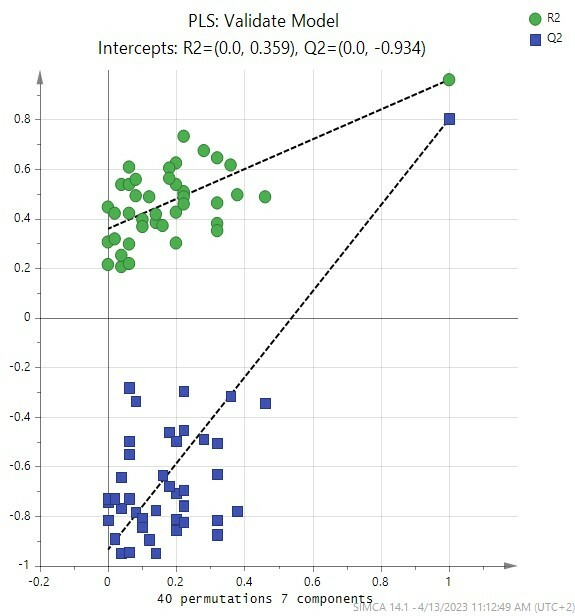


**Fig. S1.** Response permutation testing results where the vertical axis represents the R^2^Y and Q^2^Y-values of each model and the horizontal axis reflects the correlation coefficient between the ‘real’ Y and the permuted Y.
