# Supplementary material for: A voltammetric method coupled with chemometrics for determination of a ternary antiparkinson mixture in its dosage form: greenness assessment
Source: BMC Chem. 2024 May 9;18(1):90. doi: 10.1186/s13065-024-01189-0 (PMC11080133; doi:10.1186/s13065-024-01189-0)
Supplement: Supplementary file 2 — Supplementary Material 2 [file 13065_2024_1189_MOESM2_ESM.docx]

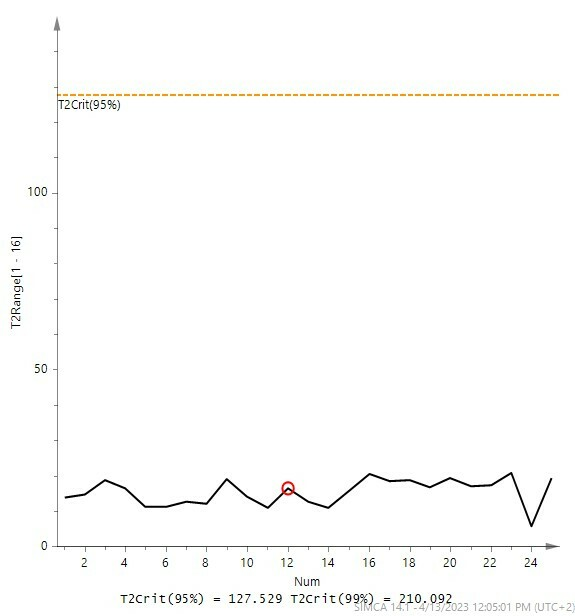


**Fig. S2.** Hotelling’s T^2^ plot, with respect to confidence limit, of the constructed PLS calibration model for LD, CD and ENT.
